# Supplementary material for: Are carbon emissions trading and green financial instruments synergistic? -Comprehensive quantitative research based on content analysis
Source: PLoS One. 2024 Mar 7;19(3):e0298601. doi: 10.1371/journal.pone.0298601 (PMC10919601; doi:10.1371/journal.pone.0298601)
Supplement: S1 Data — (ZIP) [file pone.0298601.s001.zip › Data/地级市Z_F工作报告.zip!/╜¡╬≈/╛░╡┬╒≥╩╨/└┤╫╘╣1⁄2╓┌║┼í╛┬φ┐╦╩2╛▌═°í┐_2007.docx]

各位代表：

现在，我代表市人民政府向大会作政府工作报告，请予审议，并请各位市政协委员和列席会议的同志提出意见。

　　一、2006年的工作回顾

　　过去的一年，在省委、省政府和市委的正确领导下，全市上下坚持以邓小平理论和“三个代表”重要思想为指导，全面贯彻党的十六大和十六届历次全会精神，以科学发展观统领经济社会发展全局，紧紧围绕建设“经济重镇、旅游都市、特色瓷都”的目标，开拓创新，锐意进取，扎实工作，实现了“十一五”的良好开局。

　　2006年，全市实现生产总值224.78亿元，比上年增长14.6%，三次产业结构调整为9.3：54.1：36.6，二产比重提高1.4个百分点；完成财政总收入16.3亿元，增长10.9%，其中一般预算收入9.7亿元，增长15%；城镇固定资产投资89亿元，增长0.6%；社会消费品零售总额68.1亿元，增长16.7%；城镇居民人均可支配收入9962元，同比增长13.5%；农民人均纯收入3954元，增长10.16%；城镇新增就业人员2.27万人，城镇登记失业率3.5%；人口自然增长率控制在7.76‰以内。

　　对外开放水平不断提升。组团参加了第三届“9+2”泛珠三角经贸合作洽谈会、首届中博会和文博会、香港经贸合作活动周、第四届赣台经贸合作研讨会等8项重大活动，扩大了千年瓷都的影响，签约了一批重大项目。积极转变招商模式，创新招商举措，派出60余个招商小分队，赴苏、浙、沪、粤、闽等地驻点招商，取得较好成效。2006年，全市实际利用外资5938万美元，增长16.4%；实际利用内资48.8亿元，完成年度目标101.6%，其中工业项目数和实际到位资金分别占总数的76.42%和57%。对外贸易形势喜人, 外贸出口总值1.2亿美元，增长36.9%。出口产品结构优化，陶瓷、机电、医药化工三大类齐头并进。海关、商检通关条件日益改善，“属地申报、口岸验关”作业模式全面实施，通关效率不断提高。

　　经济重镇建设积极推进。一是工业化进程继续加快。完成工业增加值95.7亿元，增长20%，其中规模以上工业增加值65.3亿元，增长25.79%。地方工业继续快速增长，完成规模以上地方工业总产值126.23亿元，增长26.7%，占全市工业比重62.2%，比上年提高2个百分点。部省属工业生产止跌转升，完成工业总产值76.68亿元，增长7.2%，同比加快4.7个百分点。园区工业增长势头良好，全市工业园区完成工业总产值59.18亿元，占全市工业比重的29.2%，增长35.27%，增速比全市快14.73个百分点。企业效益进一步好转，全市规模以上工业企业完成销售收入197.44亿元，增长33.6%，实交税金9.03亿元，增长36.2%。二是陶瓷产业不断做强。完成陶瓷工业总产值32亿元，增长25.6%；陶瓷出口创汇3793万美元，增长57％，再创历史最好水平。陶瓷工业园区按照国家高新技术开发区的标准进行规划建设，进展顺利，被省政府批准为省级开发区，台湾海畅、浙江三雄、潮州东富盈、清华大学新纪元等30余家企业入园办厂。陶瓷科技发展势头强劲，承担和主持了国家重点科技攻关项目、部省项目50多项。成功举办了2006景德镇国际陶瓷博览会，参展商、采购商数量及招商引资、内外贸总额均比上届有较大增长。规范陶瓷市场展销、梭式窑安全生产整治等工作取得实效。三是农村经济稳步发展。认真落实各项支农惠农政策，全年共发放粮食直补、综合补贴和良种补贴3400万元，农机补贴180万元，有效地促进了粮食生产，全市粮食总产达到49.8万吨，为我市历史上第二个丰产年。农业产业化步伐加快，各类农业产业化组织达到302个。农业科技进步加快，超级稻快速推广，率先在全省实施茶机购机补贴政策，农机作业水平位于全省前列。农产品质量安全水平稳步提升，重大动植物疫病防控成效显著。农业基础设施建设进一步加强，实施了镇桥联圩除险加固等一批重点水利工程，加固病险水库43座，完成中型机电排灌站更新改造3座，解决农村饮水安全人口2万人，兴建沼气池2100口。大力实施林业生态工程建设，完成春季人工造林4.21万亩，发放退耕还林粮款补助1600万元。新建改造农村公路456.5公里，行政村道路硬化率达82％。农村劳务经济加快发展，外出务工人员比上年增加2万人，务工总收入突破10亿元。社会主义新农村建设试点工作扎实推进，122个试点自然村全面完成规划编制，村庄整治工作取得实效，村容村貌得到较大改观。积极改善农村用电条件，“户户通电”工程全面完成。

　　旅游都市建设进展加快。接待国内外游客667.24万人次，增长13.9%，实现旅游总收入25.1亿元，增长19.3%，旅游总收入占生产总值的比重达11%，实现旅游外汇收入1903万美元，增长20.73%。旅游业带动直接就业人数7000余人，间接就业人数达3.5万余人。景区景点建设进一步加快，深度挖掘陶瓷文化博览区等陶瓷文化旅游产品，加强了创建国家A级景区和创建国家工业旅游示范点的申报工作，完善了高岭-瑶里、月亮湖等旅游景区景点的配套设施，整理打造金竹山寨景区。旅游服务水平不断提升，全省星级饭店服务技能大赛再获团体总分第一名，新增具有国家导游员资格人员47人，累计达到374人。成功举办了第三届茶文化旅游节和瓷文化旅游节。旅游推介取得新成效，我市被评为“中国最值得外国人去的50个地方”之一。

　　特色瓷都基础不断夯实。城市规划进一步完善，在抓紧修编城市总体规划的同时，启动或完成了城市中小学布点、住房建设（2006-2010）、城市绿地系统、城市消防、历史文化名城保护等专项规划，城区详规编制率达88.2%。重点工程建设加快，景东大道跨皖赣铁路立交桥贯通，白鹭大桥、城市防洪及一江两岸改造建设进展顺利。人民公园周边改造项目进入实质性阶段。旧城改造成效显著，斗富弄、戴家弄街区改造项目进展加快，戴家弄已完成拆迁总量的95%，斗富弄一期工程全面动工。对珠山东路、广场北路等30条主次干道进行了沥青路面和下水道维修，对小花蔑弄、太平巷等37条里弄路面进行了改造。新建旅游公厕5座，基本实现市区旱厕到水冲式公厕的改造。新建3座垃圾中转站，设置300多只分类果壳箱，市区生活垃圾无害化处理率达99.7%。在全省率先推行低保特困户免费赠卡、优惠乘坐市内公交车。新建昌江之水、春苗、高岭石、马踏飞燕等一批城市雕塑景观。建成区绿化覆盖率达41.3%、人均公共绿地面积9.31平方米，实现创建“国家园林城市”目标。杭瑞高速景德镇段顺利通车，济广高速景鹰段建设迅速， “十”字型高速公路主骨架初步形成，我市区位优势日渐显现。

　　社会事业全面进步。科技进步不断加强，“国家知识产权试点城市”建设工作进展顺利，国家日用及建筑陶瓷工程技术中心与省、市陶研所等科研院所的整合方案已经确定，中国陶瓷知识产权信息中心在我市正式成立并运行，国家陶瓷产品检测中心落户景德镇的工作正在加紧运作。组织申报国家、省级各类科技计划项目60项，已立项43项，争取国家和省科技经费659.7万元，专利申请量164件。组织鉴定验收科技成果24项，2项科技成果分别荣获2006年度江西省科技进步一等奖和二等奖。乐平被列为国家科技富民强县（市）试点县。高新技术产业进一步发展，新增国家级高新技术企业1家、省级高新技术企业3家、民营科技企业10家。教育事业加快发展，优质高中建设工程进展顺利，中小学危房改造成效显著，农村教育信息化建设加快。巩固了以县为主的农村义务教育管理体制，城乡义务教育均衡发展，职业教育规模扩大，高中阶段教育发展迅速，民办教育健康发展，老年教育富有成效。资助贫困生长效机制进一步健全。文化事业长足进步，文艺创作繁荣发展，群众性文化活动蓬勃开展，对外文化交流进一步扩大，民营文化企业发展迅速，娱乐服务场所健康有序。文物保护利用形势喜人，御窑遗址被定为国家重点文物保护单位，“景德镇手工制瓷技艺”、“景德镇传统瓷窑作坊营造技艺”被列入国家第一批非物质文化遗产名录。全面完成有线电视传输网改造，有线电视频道增加到40套，农村广播电视无线覆盖工程基本完成，自然村广播电视“村村通”工程顺利实施。公共卫生体系加快完善，市急救中心、浮梁县传染病区、浮梁疾控中心、昌江疾控中心已投入使用。改扩建乡镇卫生院9个，新建社区卫生服务中心8个、社区卫生服务站16个。人口计生工作取得良好成绩，综合考评列全省第三名，浮梁县、昌江区获全省计划生育优质服务先进县称号。体育事业蓬勃发展，群众体育运动广泛开展。获得省十二届运动会金牌数、总分数均为第三名的好成绩，金牌数和奖牌数均创历史，我市运动员在十五届亚运会上获得三枚金牌，居全省第一名。民族宗教、外事侨务、新闻出版、老龄、妇女儿童、残疾人、人防、统计、档案、地方志等各项工作也都取得了新的进步。

　　和谐社会建设成果丰硕。就业和再就业工作深入推进，新老政策实现平稳过渡，国企下岗职工基本生活保障制度向失业保险制度并轨工作年任务超额完成，下岗失业人员再就业1.17万人，困难群体再就业1073人。社会保障体系进一步完善，基本养老保险新旧制度顺利衔接，全市6.36万名企业离退休人员按时足额领取养老金。城市低保实现动态管理，享受城市低保人数达5.68万人，发放低保金4364万元。农村低保全面启动，享受低保人数3.25万人，发放低保金319万元。农村“五保户”供养标准提高，集中供养率上升到75%。城乡医疗救助制度进一步健全，救助对象达21387人（其中农村19822人），发放医疗救助款355万元（其中农村152万元）。新型农村合作医疗试点进展顺利，参合率88.24%，超出全省平均数。群众居住条件明显改善，城镇居民人均房屋使用面积达20.37平方米，农村居民人均住房面积达37.75平方米。向符合条件的困难家庭提供了一批廉租住房，向低保特困人员发放廉租补贴32万元，建设了100套廉租住房，经济适用住房面积达到10万平方米，占总竣工房屋面积比重的16.7%。优抚安置政策全面落实，获得省“双拥模范城”创建五连冠。新建农村饮水工程69处，极大改善了农村饮水条件。切实加强食品药品监管，有效地保障了群众的健康安全。加强安全生产监督管理，加大重点领域、重点行业、重点环节专项整治，安全生产总体形势平稳。切实加强社会治安工作，继续推进社会治安防控体系建设，深入开展“打黑除恶”和严打斗争，社会治安形势总体平稳，刑事案件立案数及治安案件均有所下降。以专项治理商业贿赂和解决损害群众利益的突出问题为重点，反腐倡廉工作取得新进展。

　　依法行政水平不断提高。认真落实国务院《全面推进依法行政实施纲要》，扎实推行行政执法责任制。行政行为逐步规范，行政决策进一步制度化、民主化、法制化。强化应急管理，相继制定《景德镇市突发公共事件总体应急预案》及21个专项应急预案等一系列文件。对城市建设、房地产市场、税费减免等实行规范化管理，做到有规可循、有章可依。行政服务措施进一步完善，对保留的行政许可项目面向社会公开。认真执行市人大及其常委会的决议、决定，自觉接受人大及其常委会、政协和各民主党派、工商联的监督，及时办理人大代表意见、建议59件，政协提案222件。不断优化发展环境，建立了外商投诉问题集中处理协调机制，全面开展政务环境评议评价活动和投资环境集中整治活动，营造“亲商、富商、安商”氛围。

　　各位代表，过去的一年，我们顺利实现了“十一五”的良好开局，保持了经济社会又好又快发展的势头。这些成绩的取得，是省委、省政府和市委坚强领导的结果，是全市人民团结奋斗、辛勤劳动的结果，也是与历届政府打下的扎实基础分不开的。在此，我代表市人民政府，向全市广大工人、农民、知识分子、干部和离退休老同志，向各民主党派、工商联、无党派人士和社会团体，向驻市解放军、武警官兵和公安干警，向所有关心、支持瓷都发展的海内外友好人士，表示衷心的感谢，并致以崇高的敬意！

　　在看到成绩的同时，我们也清醒地看到经济社会生活中还存在不少困难与问题。主要是经济总量仍然偏小，产业结构还不够合理，开放程度还不高，部分群众生活较困难，影响社会和谐稳定的因素仍然存在，政务环境有待进一步优化等。对于这些困难和问题，我们将高度重视，并通过发展在今后的工作中努力克服和解决。

　　二、2007年工作目标和主要措施

　　2007年是深入贯彻落实科学发展观、积极推进和谐社会建设的重要一年，是落实市第九次党代会精神、在新的起点上实现瓷都新跨越的第一年，也是为顺利完成“十一五”规划奠定良好基础的重要一年。今年政府工作总的指导思想是：以邓小平理论和“三个代表”重要思想为指导，认真贯彻党的十六大及十六届历次全会、省第十二次党代会和市第九次党代会精神，全面落实科学发展观，在市委的领导下，紧紧围绕建设经济重镇、旅游都市、特色瓷都的发展构想，以大开放为主战略，以新型工业化为核心，以“3199工程”为抓手，增加投入，做大总量，深化改革，盘活存量，加快建设融千年古韵与现代气息为一体的创新创业瓷都、绿色生态瓷都、和谐平安瓷都，推动经济社会又好又快发展，为建设富裕文明民主和谐美丽的景德镇而努力奋斗。

　　综合考虑各种因素，2007年全市国民经济和社会发展的主要目标是：全市生产总值比上年增长13%，财政总收入增长15%，其中一般预算收入增长15%，全社会固定资产投资增长20%，社会消费品零售总额增长16%，外贸出口总值增长20%，实际利用外资增长15%，实际利用内资增长15%，城镇登记失业率控制在4.5%以内，城镇居民人均可支配收入增长10.4%，农民人均年纯收入增长7.3%，人口自然增长率控制在8‰以内。

　　实现上述目标，必须切实加大投入，加速做大总量，夯实发展基础，重点实施好“3199工程”，即：在工业、陶瓷、交通、能源、农业等方面，突出抓好31个重点项目，推进经济重镇建设取得新突破；在旅游产业发展方面，突出抓好9个重点项目，推进江南旅游都市建设取得新进展；在城市建设方面，突出抓好9个重点项目，推进特色瓷都建设取得新成绩。同时，协调推进社会各项事业，构建和谐社会，促进经济社会全面协调可持续发展。

　　（一）坚持以人为本，积极实施“民生工程”

　　把关注民生作为构建社会主义和谐社会的首要任务来抓，牢固树立群众利益无小事的观念，解决好人民群众最关心、最直接、最现实的利益问题，努力实现“四个全覆盖”：城乡困难群众最低生活保障全覆盖，城乡困难群众大病医疗救助全覆盖，城乡义务教育免学杂费和贫困生资助政策全覆盖，到2008年实现农民和缺乏基本医疗保障的城镇居民合作医疗全覆盖，突出“六个重点”，办好50件实事。

　　突出抓好就业工作。主要办好10件实事：完成城镇职业培训1.7万人；发放小额贷款6800万元；农村劳动力转移培训3.68万人；劳动合同签订率92%；城镇新增就业2.16万人；跨省劳务输出人数19万人；新增转移农村劳动力2万人；“4050”人员就业900人；零就业家庭安置率达100%；城镇就业率达94%。

　　完善社会保障体系。主要办好7件实事：城镇职工参加基本养老保险人数（含离退休）25.93万人，其中农民工0.5万人，城镇大集体企业职工6.7万人，工业园区职工0.2万人，全市养老保险参保率60.9%；养老保险基金征缴总额3.52亿元；城镇职工失业保险参保人数12.9万人；城镇职工工伤保险参保人数16.1万人；农民工参加工伤保险1.8万人；完善低保制度，全年农村低保救助人数2.6万人，城市5.68万人；提高农村五保户集中供养水平，集中供养率80%。

　　努力促进教育公平。主要办好6件实事：加强农村义务教育控辍保学工作，确保农村小学适龄儿童入学率100%，年辍学率小学控制在1%，初中控制在3%；对城乡义务教育全面实施免学杂费，受益学生达18万人；为贫困生免除教科书费，受益人数3.2万人；为贫困生补助寄宿生活费，受益人数7450人；资助普通高中贫困家庭学生2166人、中等职业教育贫困学生718人、考入大学的贫困生640人；建立农村义务教育阶段中小学校舍维修改造长效机制，加大农村危房改造资金投入。

　　积极改善群众生活条件。主要办好15件实事：建设经济适用房面积10万平方米，受益1250户，廉租住房受益842户；整修老城区50条里弄路面；实施第四水厂和黄泥头水厂取水口排污整治工程；实施瓷都大道西段改造，整修沿江东路、沿江西路、中华北路、中山北路路面；新建官庄、古城等农贸市场；建设9座农村区乡汽车站和89个候车亭；新建3个垃圾中转站、6个星级公厕，新增500个垃圾箱、1000个果壳箱、5000盏路灯；改造韭菜园、向阳岭等地供水管网，解决2000户居民饮水难问题；搬迁第五中学，解决一校三址问题；抓好223个新农村建设试点自然村，并扩充试点内容；新建20处农村饮水工程，解决2.1万农村人口饮水难问题；新增农村沼气池4000口；完成200个20户以上自然村广播电视“村村通”工程，完成乐平市草鞋山发射台和浮梁县程山发射台第二期广播电视无线覆盖工程；改善农村群众出行条件，投资1.32亿元新建改造农村公路440公里，行政村道路硬化率达90%；加快实施光明电影院改造工程，解决群众看电影难问题。

　　切实解决群众看病难看病贵问题。主要办好8件实事：扩大城镇职工基本医疗保险扩参保对象，参保人数19.9万人，其中国有困难企业职工参保人数3万人，城镇职工医疗保险参保率54.43%，城镇职工基本医疗保险统筹资金使用率80%；积极推进农村新型合作医疗，将昌江区纳入新型农村合作医疗试点范围，全市参加合作医疗人数79万人，参合率85%以上，筹集合作医疗资金3953万元，资金使用率75%；推进城镇居民合作医疗，参合人数达到10万人，参合率38%，城镇大集体企业职工1.2万人；实施农村困难群众大病医疗救助制度，救助人数1.6万人，资金使用率80%；实施城市困难群众大病医疗救助制度，救助人数9000人以上，资金使用率80%；加强农村卫生机构建设，确保141个村卫生室（所）达标，15个乡镇卫生院达标；积极完善社区卫生服务网络，确保12个社区卫生服务机构达到建设标准；开工建设市妇幼保健院新病房大楼，提高妇幼保健水平。

　　不断提高群众的收入水平。在发展经济的基础上，进一步改革和理顺收入分配关系，千方百计提高中低收入者的收入水平，让改革和发展成果充分惠及广大人民群众。主要办好4件实事：落实各项惠农支农政策，切实巩固农民减负成果，有效增加农民收入；努力扩大就业，多渠道增加群众的就业机会；提高最低工资标准，增加城镇职工工资性收入；加强消费品市场物价监控，稳定基本生活消费品物价水平。今年要确保城镇居民人均可支配收入达到1.1万元，农民人均纯收入4244元。

　　（二）着眼又好又快，推动瓷都经济跨越发展

　　要紧紧抓住重要战略机遇期，全面贯彻落实科学发展观，坚持好中求快，以快促好，努力实现经济重镇、旅游都市和特色瓷都建设的跨越式发展。

　　第一，着力壮大经济总量，推进经济重镇建设取得新突破

　　以新型工业化为核心，实现工业经济新增长。既做顶天立地的大项目，种好“大树”，又做铺天盖地的中小项目，培育“灌木、草丛”，形成良好的“工业生态”环境。完成规模以上工业增加值80亿元，销售收入235亿元，利税总额12亿元。一是加速建设工业园区。围绕基础设施、招商引资、产业聚集、高效服务四个重点，完善供水、供电、供热、供气、污水排放等基础设施建设。高新技术产业园区要努力完成平整工业用地2000亩、投入20亿的目标，陶瓷工业园区要按照做成国内有影响的陶瓷特色园区、乐平工业园区要按照做成全省有规模的特色化工园区的要求，加快项目建设。2007年，全市工业园区规模以上工业占全市工业的比重达到30%以上。二是加快实施重大项目。突出抓好9个重大工业项目，即：昌飞扩改、昌铃汽车生产线改造、锦溪水泥日产4500吨熟料生产线、电化精细化工水合肼、电化高科AC发泡剂和氯化亚砜、天新药业维生素B6、景航锻铸高新技术及机械项目、开门子干熄焦和华意压缩经济型HYE压缩机开发等，当年完成投资8.9亿元。以大项目培育大企业，以大企业支撑支柱产业，全市七大支柱产业规模以上工业增加值占全市工业总量的比重达到90%以上。三是突出企业创新发展。推进工业技术由研发型向先进适用型转变，提高科技成果转化率。重点开发昌飞多类型直升机、昌汽系列汽车、中景机电一体化多色胶印机和对开胶印机、釉中彩瓷和工业技术陶瓷、化工行业的捣固焦和特种聚乙烯醇等一批重点新产品项目，增强自主创新能力，提高企业竞争水平。今年，要重点建设2个国家级技术中心、2个省级技术中心，完成重点新产品开发项目10个，完成重点新产品试产项目10个，力争全市工业经济效益综合指数提高10个百分点以上。

　　以产业振兴为重点，实现陶瓷经济新跨越。要以实现陶瓷产业基本振兴为目标，抓住省部共建景德镇国家陶瓷区域创新体系的机遇，努力打造产学研一体化、产供销市场化国际化的大陶瓷产业新格局，建设名符其实的“中国的瓷都、世界的景德镇”，力争全市陶瓷业总产值达到40亿元以上，陶瓷出口创汇达到5000万美元。一是做大陶瓷产业。着力扶持一批优势陶瓷企业，培育规模以上陶瓷企业70家，其中亿元以上企业3至5家。突出抓好8个陶瓷项目，即：法蓝瓷公司三期建设项目、东璟公司高档日用瓷扩建项目、东富盈公司二期建设项目、南瓷公司绝缘子项目、神飞特陶年产125万件氧化铝陶瓷、三雄陶瓷年产日用陶瓷和高档酒瓶2500万件、江西万平真空电器有限公司生产陶瓷真空电容器项目、鹏飞建陶年产61万立方米/微晶玻璃陶瓷砖生产线等，当年完成投资1.72亿元。二是推进陶瓷科技创新。启动陶瓷新材料、陶瓷信息化科技工程，加快陶瓷高新技术的消化、引进、吸收。重点攻关发光陶瓷材料、陶瓷膜技术等10个重大陶瓷科研项目，整合陶瓷教育科研资源，加速陶瓷科技成果向现实生产力转化。三是规范搞活陶瓷商贸流通。进一步培育和做大陶瓷市场，推进陶瓷销售向专业市场集中。逐步建立健全覆盖全国的陶瓷销售网点和网络，有序建立国外陶瓷销售网络。规范陶瓷市场经营秩序，引导陶瓷业主合法经营、依法纳税。继续整顿和规范陶瓷出国和异地展销秩序。围绕“一年站稳、三年站好、五年站高”的目标，全力办好2007景德镇国际陶瓷博览会。四是构建开放式、多元式、低成本的陶瓷产业发展保障体系。加强以引进高效清洁天然气为重点的陶瓷能源建设，降低生产成本。加紧陶瓷原料勘探和基地建设，推行陶瓷原料生产标准化，打造资源保障体系。提升机械装备、模具、窑炉、耐火材料、花纸金水和陶瓷包装生产质量和水平。改造现有高能耗隧道窑，推广新型隧道窑和辊道窑。大力实施品牌战略，加强“景德镇”证明商标管理，加强商标和专利保护，严厉打击假冒伪劣等侵权行为。

　　以现代农业为重点，实现农业农村新发展。坚持以发展农村生产力为中心，用工业化思维谋划农业发展，加大对农业的投入，提高农业综合生产能力，推进社会主义新农村建设。2007年，实现农业总产值增长6%，力争粮食总产突破5亿公斤。一是大力推进农业产业化。集中力量扶持德宇集团、乐平蔬菜集团、浮梁茶叶有限公司等12家国家、省级农业龙头企业做大做强，力争培育几个产值超亿元和5亿元的农业产业化龙头企业，新增省级龙头企业3家，进一步延伸我市农业产业链，提高农业效益，实现全市龙头企业带动基地面积80万亩，带动18万农户户均增收500元。二是抓好农业基地建设。继续推进农业三大体系建设，建设优质粮食生产基地。充分发挥我市乐平蔬菜、浮梁茶叶、昌江养殖等特色优势，加快构建“一乡一业、一村一品”农业特色块状经济，力争全市高效经济作物产值达到13.8亿元，占种植业比重提高3个百分点。三是加强技能培训和科技推广工作。积极推进农业科技进村入户，加快农业机械化进程。扩大“阳光工程”实施规模，提高农民的就业技能。大力推行农业标准化生产，加强无公害农产品、绿色食品、有机食品的申报认定工作，加速培植一批优质农产品品牌。四是加强农业基础设施建设。突出抓好6个农业基础设施项目，即：幸福水库、东方红水库、勤俭水库除险加固工程、镇桥联圩建设、农村饮水安全工程、小Ⅰ型水库除险加固工程、植物园建设和沼气、测土配方施肥、阳光工程培训等，当年完成投资1.13亿元。五是大力推进县域经济发展。着眼统筹城乡发展，以工促农、以城带乡，大力推进小城镇建设。充分发挥各县（市、区）的比较优势，打造特色鲜明的经济板块，加快产业聚集和区域特色经济发展。乐平市要充分发挥现有产业优势，争取在全省排名争先进位；浮梁县要在大力发展工业的同时抓好旅游、特色农业的发展，建设生态名县；昌江区要发挥城郊优势，做好配套服务文章；珠山区要立足城区，大力发展旅游、社区经济等第三产业。六是继续扎实推进社会主义新农村建设试点工作。坚持“政府主导、农民主体、干部服务、社会参与”新农村建设运行机制，规范试点内容，扩大试点规模，落实扶持资金，建设一批具有较高标准、有较强示范带动作用的新农村示范点，促进新农村建设的全面开展。

　　第二，着力提升旅游产业发展水平，推进旅游都市建设取得新进展

　　坚持以大开放的理念、大开放的战略、大开放的手段来促进新一轮的旅游大发展，进一步提高瓷都旅游的知名度、美誉度和影响力，实现发展水平的全面跨越。2007年，接待国内游客760万人次，创收27亿元；接待境外游客7万人次，创汇2284万美元。实现旅游总收入28.75亿元。

　　凸显瓷都特色，打造旅游景区。坚持保护为先、合理利用的原则，保护好历史文物，打造城区里弄景点，将文物资源开发转化为旅游资源和经济增长点，展示历史文化名城的古韵风貌。突出抓好9个旅游项目，即：御窑陶瓷历史文化旅游、景德镇游客集散中心、景德镇国家森林公园德宇龙山园、瑶里国家级历史文化名镇保护及旅游开发建设、高岭土瓷矿遗址保护工程、金竹山寨旅游区开发、紫晶宾馆、西山湖宾馆和体育中心项目等，当年完成投资3.2亿元。加强陶瓷历史文化博览区、浮梁古县衙等王牌旅游景区建设。在市区内打造以御窑为核心的民窑柴巷景区、刘家弄博物馆、吊脚楼、三闾庙等3至5个旅游新景点，加快建设陶瓷名人艺术馆，规划创建2个陶瓷工业旅游示范新景区，力争新增2个国家4A级景区，启动瑶里5A级景区创建和高岭国家矿山公园建设工作，进一步完善玉田湖、月亮湖等风景区的旅游度假功能。

　　整合旅游资源，扩大旅游投入。创新旅游发展机制，破解旅游产业投入的课题。在对景区进行有效保护的基础上，实现所有权和经营权分离，把景区（点）的经营权推向市场，按照社会化投入、市场化运作的方法，整合盘活旅游资源。加大招商引资力度，争取知名大型旅游企业进入景德镇，通过若干旅游企业的资源整合，打造旅游龙头企业。完善旅游功能建设，规范旅游购物市场和餐饮市场，着手建设景德镇市旅游网站，加快建设市区至洪岩、瑶里的旅游公路，推进瑶里通往婺源灵岩洞的道路建设工作。

　　强化推介协作，开拓旅游市场。创新旅游宣传促销手段，主攻日、韩、港、澳、新加坡、台湾等国外境外市场，构建与赣东北、武汉、广州、上海、黄山等重点国内客源市场的协作机制。重点包装宣传推介景德镇古陶瓷文化旅游资源和产品，收集整理陶瓷历史文化故事，创作反映景德镇历史文化的宣传片，争取编一个“好故事”，写一个“好本子”，拍一部“好片子”，出一台“好戏剧”。提高景区标识设置标准，标识采用中文与英文、日文、韩文对照。办好第四届瓷茶文化旅游节，打响旅游节庆品牌。组建营销中心，强化推介手段和力度。加强区域性的旅游合作，打造上海－杭州－黄山－景德镇、北京－景德镇－黄山和杭州－黄山－景德镇的旅游精品线路，重点包装“景德镇－婺源－黄山”、“景德镇－婺源－三清山”等旅游线路。

　　大力发展现代服务业，培育和发展物流市场。重点建设景德镇物流园区，争取早日发挥物流园区对我市经济发展的拉动作用。加快发展商贸业，积极推进商贸流通企业的重组工作。实施“万村千乡”市场工程，进一步完善乡、村“农家店”的硬件设施。出台我市商业网点布局规划，力争创建1-2家省级绿色市场，加快建设“景德镇农特产品批发大市场”。加快发展金融、保险等行业，进一步加大金融资金对地方经济的投入。

　　第三，着力实现传统文明与现代气息相结合，推进特色瓷都建设取得新成绩

　　按照城市向西扩展，工业向南聚集的发展格局，坚持千年古韵与现代气息相结合，护其貌、显其神、保其韵、铸其魂，打造城在山中、水在城中、人在画中的山水、生态、文化特色城市。

　　坚持科学规划。充分发挥规划龙头作用，坚持规划科学性、操作性和严肃性的统一，严格实行规划一张图，建设一盘棋，审批一支笔，管理一条龙。着手编制《景德镇市城市景观规划设计》、《景德镇市近期建设规划》、《景德镇市城镇体系规划》和《一江两岸景观设计规划》，抓紧西山片区规划、新区部分地段控制性详规和景东大道片区控制性详规的修订完善工作，全面完成城市总体规划报批工作。

　　推进城市建设。在保护历史文物、保持历史风貌的基础上，突出城市功能的完善，提升城市品味和城市形象，既体现瓷都千年文明的传承，又展现现代化都市的风貌，实现传统与现实的完美融合。突出抓好9个城市建设项目，即：韭菜园路网、城市防洪与一江两岸改造、白鹭大桥、湖田小区经济适用房建设项目、西瓜洲污水处理厂、戴家弄街区改造、斗富弄街区改造和景德大道建设等，当年完成投资6.01亿元。积极推进人民公园周边改造等项目。加快实施美化亮化工程，让街道亮起来，让昌江清起来，让城市美起来，让百姓乐起来。

　　搞活城市经营。坚持“市场运作、自求平衡、滚动发展”的经营城市理念。加强土地市场宏观调控，加快投融资体制改革，充分利用国内外资本市场，创新多元化、多渠道城市建设投融资方式，推行项目市场化运作，发掘项目“造血”机能。搞活市政基础设施经营，全面推行公用设施有偿使用、特许经营和市场化运作，努力把市政基础设施的使用权、经营权和产权推向市场。全面推开市政公用事业单位改革，开放市政公用行业市场。

　　强化城市管理。按照社会管理、经济管理、规划管理、公共建设管理和生态环境管理五大管理内容，进一步加强城市管理。从城市卫生入手，完善“车线联动”和“四位一体”城市管理模式，整治市容市貌，建立长效管理机制。加强社区建设，提升社区服务水平。整合执法队伍，加大城管执法力度，开展违法建设专项整治，严厉查处老城区和城乡结合部乱搭乱建行为。

　　深入开展创建。始终把创建工作作为不断提升城市整体形象及竞争力的抓手，坚持在高位推动上加强领导，在整体推动上落实责任，在软硬结合上突出特色，在市民参与上注重实效，进一步巩固中国优秀旅游城市、国家园林城市创建成果，做到劲头更足、力度更大、投入更多、管理更强，向更高创建目标迈进。加快创建“五城”工作，争取2007年获得第二届江西省文明城市称号，创国家卫生城市通过省级考核推荐。

　　（三）加快推进改革开放和全民创业，进一步增强经济发展活力和动力

　　深入学习“求真务实、诚信和谐、开放图强”的浙江精神，把扩大开放与发展内源型经济更紧密的结合起来，把“引进来”和“走出去”更紧密地结合起来，把国际国内两个市场、两种资源更好地利用起来。充分发挥我市的区位优势、资源优势和后发优势，开拓经济发展空间，集聚经济发展外源活力；适应市场取向改革的发展趋势，坚定不移地走全民创业发展之路，激活经济发展内源动力。

　　坚定实施大开放主战略。2007年，全市2000万元以上工业和5000万元以上综合类重点项目招商引资完成56亿，其中5000万元以上工业项目引资占到总数的50%以上；外资完成6829万美元；外贸出口完成1.4亿美元。一是扎实做好产业招商。注重围绕产业招商选资，做长产业链，实现产业的集群化、企业的配套化、产品的链式化。重点打造汽车产业链、陶瓷产业链、医药化工产业链和电子信息产业链。二是突出重大项目招商。瞄准国际国内大集团、大公司，尽快引进一批上规模、上档次、带动能力强的重大项目，培育一批骨干龙头企业。深化香港新世界与电化高科的合作，做好乐华、金意陶、欧神诺等大型陶瓷企业落户服务工作，争取元九国际开发公司来我市发展。三是突出重点地区招商。重点抓好对长三角、珠三角、台港澳的招商，主动承接沿海产业转移，尤其要加强对浙江的招商工作。四是不断扩大对外贸易。扩大汽车及零部件、陶瓷、机电、医药化工等产品的出口规模。扩大外资企业和民营企业出口份额，增强国有企业出口能力。推进企业发展自主出口品牌和销售网络。五是打造快速便捷交通网络。突出抓好5个交通项目，即：景德镇至鹰潭高速公路、南环高速湘湖至丽阳段、湘湖至官庄一级公路改造工程、吴乐秧等战备公路工程和农村公路改造工程等，当年完成投资25.5亿元。

　　深入推进全民创业。大力培育创业文化，放宽民间资本准入领域，拓展民营经济发展空间，积极引导民间资金从储蓄转向生产、从消费走向生产，在全市上下形成鼓励、支持和引导民营经济发展的良好氛围。增加创业资金投入，提高财政对创业资金扶持比例。鼓励金融机构出台支持创业政策，做大中小企业担保金总量，力争市中小企业信用担保资金总量达到1亿元以上。进一步激活各类创业主体，支持经营管理者和科技人员领头创业，吸引外出务工人员返乡创业，扶持下岗失业人员自主创业，推动农村基层干部和群众致富创业，鼓励干部在富民兴市中干出一番事业，真正形成“百姓创家业、能人创企业、干部创事业”的创业大潮。

　　深化重点领域改革。进一步建立健全国有资产监督管理体制，由市国资委履行国有资产出资人职责，指导、推进全市国有企业的改革和重组，加强国有资产管理，推动国有经济结构和布局的战略性调整。深化国有企业改革，采取合资合作、增资扩股、产权转让等形式，积极引进国内外有实力的战略投资者，推进产权多元化，深化国有企业股份制改革，建立现代企业制度。充分研究、利用好国家促进中部崛起的有关政策，认真做好打捆回购不良资产工作，推进全市国有企业债务重组，切实做好国有困难企业改革脱困工作，依法积极稳妥推进企业政策性破产工作。进一步深化金融体制改革，不断推进银企合作，改善金融服务，防范金融风险，加快城市信用社组建城市商业银行步伐。认真落实工资改革和规范津贴补贴工作，扎实推进财政改革，全面推行部门预算，推动“收支两条线”完全脱钩试点，深入研究完善“乡财县代管”改革试点，规范非税收入管理。推进农村综合改革，重点抓好乡镇职能转变和人员分流，做好化解乡村债务试点工作，统筹推进畜牧兽医管理体制、种子管理体制和农技推广服务体系改革，继续完善林业产权制度配套改革。进一步规范土地承包经营权流转，促进农业规模化经营。

　　（四）加快资源节约型环境友好型社会建设，进一步增强经济可持续发展能力

　　倍加珍惜宝贵的资源，倍加珍惜良好的生态环境，以创建国家环境保护模范城市为抓手，推行有利于资源节约和环境保护的生产模式、消费模式和城乡建设模式，使瓷都青山常在、绿水长流、资源永续利用。

　　切实加强资源节约和综合利用。突出抓好3个能源项目，即：乐平矿务局煤矸石发电厂设施建设、樟树坑水电站和乐平矿务局采煤沉陷区综合治理基础设施建设等，当年完成投资4.18亿元。加大电网改造建设力度，加强电力设施保护。扎实开展节能降耗，重点抓好高耗能企业节能、房屋建筑工地（小区）节能和政府建筑物、办公系统节能改造，引导商业和民用节能，大力推进节水、节电和节材工作。抓好企业清洁生产工作，支持清洁生产技术开发、示范和推广工作。加强资源综合利用，拓宽资源综合利用行业和领域，提高废渣、废水、废气、余热、余压的综合利用率。开展“国土资源管理年”活动，大力推进土地集约利用，严格土地准入管理。加强对基本农田保护区的保护，把管地、造地、用地有机结合起来，切实落实耕地占补平衡，稳定全市耕地总量。抓好矿产资源的合理开发和综合利用。加强矿产资源勘查、开采、使用有序管理，限制和淘汰粗放型资源开采、经营项目。

　　强化环境保护。严把环境保护准入关，切实做到严重污染环境的项目坚决不搞；严重危害人民生命健康和职工安全的项目坚决不搞；黄、赌、毒的项目坚决不搞。对超过污染物排放总量控制指标、生态破坏严重的区域，停止审批新增污染物排放的建设项目；对污染处理设施不能稳定达标或超总量排污的企业，坚决责令整改，直至关闭。严格控制排污，实行排污许可证制度，确保全年二氧化硫排放量削减0.2万吨、化学需氧量排放量削减0.05万吨。抓好固体废物综合利用和危险废物管理，提高资源综合利用率。进一步保护好水资源，确保集中饮用水水源水质达标率达到100%。

　　加强生态建设。坚持保护优先、开发有序，以控制不合理的资源开发为重点，强化对水源、土地、森林等自然资源的生态保护。严格依法保护昌江河、乐安河等重要水源保护地区，确保人民群众有纯净卫生饮用水。加强天然林保护，落实好生态公益林的补偿政策，适当提高补偿标准。严格控制森林特别是严禁天然阔叶林的商业性采伐，做好封山育林工作，坚决打击偷伐、偷运。抓好植树造林，力争全年完成植树造林面积10万亩。加强水土流失保护，搞好小流域治理，继续实行退耕还林，减少人为水土流失，保护好水生生物。加大河道整治力度，维护河道安全、通畅。加强乐平矿区生态环境综合治理。建立健全生态环境投资补偿运行机制，为经济社会可持续发展提供保障。

　　（五）注重协调发展，实现社会事业全面进步

　　以经济社会协调发展和人的全面发展为目标，大力推进科教兴市、人才强市和可持续发展战略，统筹发展各项社会事业，妥善化解各类社会矛盾，以发展求和谐，以和谐促发展。

　　坚持教育优先方针，大力发展教育事业。巩固提高两基成果，全市小学适龄儿童入学率达100%。大力发展中等职业教育，在高中毛入学率达到65%的基础上，职业高中招生6200人，职普比例达到4：6。积极争取农村远程教育工程资金，力争农村远程教育覆盖率100%。进一步完善城区中小学布局。继续实施2006－2007年全省农村中小学基础设施改造工程。抓好市一职实训基地、市三中扩建、陶艺基地二期等项目建设。完成市直中小学教职工全员聘用合同制改革。发展高等教育，进一步支持景德镇陶瓷学院的建设和发展，加快推进景德镇学院组建工作。继续关心和支持老年人教育事业发展。

　　加快推进科技进步，增强自主创新能力。坚持“自主创新，重点跨越，支撑发展，引领未来”的方针，以加快经济发展为主题，以平台建设为基础，以项目建设为支撑，以提升综合科技水平为目标，完善科技服务体系，提高科技创新能力，加快科技进步步伐。围绕建设创新型城市，在推进国家日用陶瓷及建筑陶瓷工程技术研究中心、市高新技术产业园区和陶瓷工业园区建设的基础上，重点抓好国家火炬产业化基地申报、国家级高新技术产业开发区申报的衔接工作，承办2007中国景德镇高技术陶瓷国际论坛。新增省级以上高新技术企业3家，专利申请量达到165件，组织申报国家、省级科技计划项目65项。实施星火富民工程、陶瓷新材料工程、制造业信息化工程三大科技工程。

　　大力弘扬先进文化，丰富群众文化生活。依托丰富的陶瓷文化内涵、众多的历史文化古迹、多姿多彩的民间陶瓷文化活动，做强“三大品牌”，推出“四大工程”，完成“五个一批”，即：做强精品文化品牌、群众文化品牌、文化产业品牌，推出文化先进县创建工程、文化信息资源共享工程、非物质文化保护工程、文化精品下基层工程，打造一批时代精神昂扬、地域特色鲜明的文艺精品，建设一批功能完善、服务优良的标志性文化设施，举办一批规格较高、具有一定号召力和影响力的大型文化活动，培育一批主业突出、多业发展、运营规范、效益明显的文化骨干企业，培养一批德艺双馨的专业技术人才。

　　加快发展卫生体育事业，提高群众健康水平。加强公共卫生体系建设，健全突发公共卫生事件应急机制，重点抓好市医疗紧急救援中心、市传染病医院的组建工作，切实抓好重大疾病的预防控制。进一步加强农村卫生工作，实施县级综合医院、乡镇卫生院、村卫生所等房屋和设备建设项目。加快发展社区卫生服务事业，方便社区居民看病。申请增加一个省级训练基地，增加若干社区群众体育活动场所。加强人口与计划生育工作，控制人口数量，提高出生人口素质，切实降低出生人口性别比。

　　扎实做好安全稳定工作，构建和谐平安瓷都。进一步落实安全生产责任制和安全事故行政责任追究制度，严防重特大安全生产事故发生。加强各种自然灾害预测预报，提高防灾抗灾减灾能力。进一步加强食品药品监管工作，保障人民群众饮食、用药安全。积极推进社会治安综合治理，健全矛盾排查调处机制；运用宽严相济的刑事政策，深入开展“严打”整治斗争；完善社会治安防控体系。认真做好信访工作，建立健全群体性事件预警机制、突发公共事件应急机制，深入开展平安创建活动。进一步做好“双拥”工作，继续创建全国双拥模范城，不断巩固和发展军政军民团结。

　　三、加强政府自身建设

　　按照建设“和谐、创新、务实、高效、自律”政府的要求，各级政府和领导干部必须适应新形势新任务，不断提高行政能力和管理水平。

　　转变政府职能, 推进管理创新。按照政企分开、政事分开、政府与市场中介组织分开的原则，继续深化行政管理体制改革，把心思放在百姓身上，把功夫下到百姓身上，使政绩体现在百姓身上，更加注重履行社会管理和公共服务职能。坚持市长手机短信号码公开制度，帮助群众排忧解难。创新公共服务体制，加大公共服务支出，为全市人民提供更多更好的公共服务。认真贯彻《行政许可法》，继续深化行政审批制度改革，健全行政服务中心职能，简化审批程序，提高行政审批效率。切实提高行政效能，增强政府执行力和公信力。继续推进电子政务建设，完善“一站式办理”、“一条龙服务”、限时办结等制度。加强社会信用体系建设，完善行政审批责任制和过错责任追究制，继续推行行政机关服务承诺制度。加快建立企业信用评价、监督管理、责任追究等信用制度，促进企业守法经营。加强诚信教育，提高公民诚信意识，加大失信惩戒力度，依法维护社会公正。

　　完善决策机制，提高决策水平。积极推进科学决策、民主决策，合理界定政府的决策权限，进一步完善公众参与、专家论证和政府决策相结合的决策机制，保证决策的科学性和正确性。加快建立和完善重大问题集体决策制度、专家咨询制度、社会公示和社会听证制度、决策责任制度，重大问题注重听取市人大、市政协意见，征求党委意见并报备案，提高决策的民主化、科学化、法制化水平，使决策更具实际效果和可操作性。

　　坚持依法行政，规范行政行为。继续加大《全面推进依法行政实施纲要》的贯彻实施力度，规范行政执法行为，强化对执法部门的监督。严格依照法定权限和程序行使权力、履行职责、接受监督，依法界定和规范经济调节、市场监管、社会管理和公共服务的职能。进一步明确行政执法权限，减少行政执法层级，完善执法程序，提高执法水平。完善行政复议、行政赔偿和补偿等制度，加大行政综合执法改革力度，加快推进相对集中行政处罚权的改革工作。整合执法资源，坚决克服多头执法、执法不公，甚至执法违法等现象。建立健全行政执法案卷评查制度，进一步规范行政处罚行为。加强行政执法监督工作，积极开展法律法规实施的跟踪检查。落实行政执法责任制，完善行政层级监督机制，真正做到有权必有责，用权受监督，侵权要赔偿，违法要追究。认真执行市人大及其常委会的决议、决定，自觉接受人大及其常委会的法律监督和工作监督，自觉接受政协和各民主党派、工商联的民主监督，及时办理人大代表议案、建议和政协委员提案，积极加强沟通，争取支持，自觉接受人民群众的社会监督和新闻媒体的舆论监督。

　　加强队伍建设，严格廉洁自律。认真贯彻《公务员法》，加强公务员能力和素质建设。完善公务员学习、培训和考核制度。加强公务员思想、政治、作风建设，增强公务员的大局意识、责任意识、创新意识和服务意识。加强政风行风建设，以规范行政权力为重点，健全教育、制度、监督并重的惩治和预防腐败体系。进一步完善权力运行监督机制，强化执法监察和审计监督。依法严肃查处在工程建设、产权交易、政府采购等领域的商业贿赂案件和损害群众利益行为。严格执行工程质量终身责任追究制。对涉及行政许可、行政收费、专项资金、大型工程、土地使用、城镇拆迁、企业改制等关系人民群众切身利益，同时又容易出现腐败问题的事项，坚决向社会公开，让群众监督。不断推进制度建设和创新，不断提高政府机关公务员依法行政的观念和依法处理经济社会事务的能力，防止权力失范失衡导致腐败问题的发生。

　　各位代表，今年是贯彻落实省第十二次党代会和市第九次党代会精神，努力在新的起点上实现瓷都新跨越的开局之年，让我们紧密团结在以胡锦涛同志为总书记的党中央周围，全面落实科学发展观，在省委、省政府和市委的领导下，与时俱进，团结拼搏，扎实工作，为打造富裕民主文明和谐美丽的新瓷都，实现经济重镇旅游都市特色瓷都建设新跨越作出更大贡献！
